# Hope Across Socioeconomic Status: Examining Measurement Invariance of the Children’s Hope Scale Across Socioeconomic Status Groups

**DOI:** 10.3389/fpsyg.2019.02593

**Published:** 2019-11-21

**Authors:** Hui Lei, Zhihang Wang, Ze Peng, Yanyun Yuan, Zhihua Li

**Affiliations:** ^1^College of Education, Hunan Agricultural University, Changsha, China; ^2^College of Teacher Education of Ningbo University, Ningbo, China

**Keywords:** CHS, hope, measurement invariance, socioeconomic status, SES

## Abstract

There has been a growing interest in research on hope in recent years. The Children’s Hope Scale (CHS) is the most commonly used scale to evaluate goal-related hopeful thinking in children and adolescents. Socioeconomic status (SES) strongly influences an individual’s experiences from childhood and throughout adult life. This study aimed to evaluate the measurement invariance of the CHS across SES. The sample consisted of 1934 Chinese youths (50.4% females) with a mean age of 12.96 (SD = 2.686). An overall family SES score was obtained by totaling the *Z* scores for family monthly income and parents’ education level. The results supported the single-factor model as the baseline model across each SES group. Multi-group confirmatory factor analysis revealed that full measurement invariance did not hold. One factor loading and one intercept were non-invariant. There were also significant differences in latent factor means and raw scores of the CHS across the two groups. The CHS had a stronger convergent validation in the higher SES group than lower SES group. The results suggest that researchers and practitioners should exercise caution when comparing differences in hope measured by the CHS between groups with different SES. We provide more robust statistical evidence in terms of SES differences, indicating that children and adolescents from higher SES backgrounds shower greater hopeful thinking compared with those from lower SES backgrounds.

## Introduction

The concept of hope has received increasing attention in recent years from various perspectives, including psychology, psychiatry, philosophy, religion, and mythology, among others. Hope is viewed as “a positive motivational state” (Snyder et al., 1991b), and is associated with positive events that individuals believe will happen in the future and has been viewed as an important predictor of positive outcomes (Snyder et al., 2002; Park et al., 2004; Edwards et al., 2006; Creamer et al., 2009). Psychologists have attempted to conceptualize and measure hope in order to better understand its influence on the development of children in recent decades.

Hope has been defined in earlier literature as a unidimensional construct referring to a general perception that one’s goals can be met (French, 1952; Stotland, 1969). Snyder et al. (1991a) defined hope as “goal-directed thinking in which the person has the perceived capacity to find routes to goals (pathways thinking) and the motivation to use those routes (agency thinking).” They proposed the hope model, which included three distinct components: goals (providing the target of mental action sequences, which need to be of sufficient value to occupy conscious thought), pathways (signifying one’s perceived ability at generating workable routes to desired goals), and agency (the perceived ability to use one’s pathways to achieve desired goals) (Snyder et al., 2002, 2003).

The Children’s Hope Scale (CHS) was developed by Snyder et al. (1997) to assess hopeful thinking in children and adolescents. The CHS is the most commonly used measure of hope. It sparked significant research on children’s hope and its correlates. Specifically, children with higher levels of reported hope tended to have more global life satisfaction (Valle et al., 2004); better psychological well-being (Snyder et al., 2000; Merkaš and Brajša-Žganec, 2011); higher-rated physical appearance, enhanced self-perception of athletic ability, greater scholastic competence and social acceptance (Snyder et al., 1997); quality of life (Martins et al., 2018); and greater academic achievement (Dixson and Stevens, 2018). They may also have lower levels of depression (Snyder et al., 1997) as well as decreased externalizing and internalizing behavioral problems (Gilman et al., 2006). Hope has also been reported to be a significant predictor of multitudinous positive outcomes in children and adolescents, such as academic achievement (Dixson and Stevens, 2018) and quality of life (Martins et al., 2018).

The initial study provided psychometric evidence in support of the CHS (Snyder et al., 1997). The psychometric properties of the CHS were reported not only in American and European contexts, including the United States (Valle et al., 2004), Portugal (Marques et al., 2009), Serbia (Jovanović, 2013), and Spain (Pulido-Martos et al., 2014), but also in other national or cultural contexts, including Turkey (Atik and Kemer, 2009), China (Zhao and Sun, 2011), and South Africa (Guse et al., 2016). Measurement invariance is an important property of psychometric instruments as it is a pre-condition for assuming one scale to be equal across different groups or times. This assumption is necessary to conclude interpretable and valid comparisons of the differences in the scale’s scores (Billiet, 2002). The measurement invariance of the CHS has received increasing attention in recent years. For example, Dixson (2017) reported the measurement invariance of the CHS across the range of achievement, and Jovanović (2013) examined measurement invariance across gender. In this study, we focus on determining whether the CHS exhibits measurement invariance across socioeconomic status (SES).

Socioeconomic status, a measure of one’s general social status and position, strongly influences his/her life experiences and development from childhood through adulthood (Hackman et al., 2010). The family investment theory is a theoretical paradigm used to explain the correlation between SES and human development. The theory is based on the notion that higher SES parents have increased access to financial, social, and human capital compared to lower SES parents. In turn, the investment of these resources by families is related to the successful development of children and adolescents (Conger and Donnellan, 2007). Empirical studies show that adolescents with higher family SES have better social identity and increased levels of happiness (McAuley, 2012). Compared with children and adolescents from higher-SES backgrounds, children and adolescents from low SES backgrounds show worse cognitive and emotional development, social adaptation, health, and psychological well-being (McLoyd, 1998; Bradley and Corwyn, 2002; Plenty, 2018). Family SES is also associated with the orientation and planning of adolescents’ future goals. Adolescents growing up in a low SES family tend to have a more negative outlook and unclear expectations of their future compared to those adolescents growing up in a high SES family (Griskevicius et al., 2011; Schroder et al., 2011). We also found family SES to have a significant effect on the developmental trajectory of hope among adolescents (Yin et al., 2019). These findings provided evidence that SES has a substantial impact on the development of child and adolescent hope.

As the most populous developing country, China is at a particular stage of development. In the last several decades, China’s GDP has changed greatly alongside a widening of the income gap among Chinese residents, ultimately leading to a change in overall family SES (Bao et al., 2002; Molero-Simarro, 2017). Socioeconomic inequality in China has been gradually rising in recent decades and is drawing more attention. Recent studies have shown that this rise in socioeconomic inequality may influence Chinese children’s education and mental health. It has been shown that family SES is correlated with Chinese children’s academic achievement (Liu et al., 2019). Students from higher SES families are able to acquire more extensive social and educational resources to access high-quality schools, leading to greater academic success (Ding and Lu, 2005; Feng and Lu, 2010). Higher family SES increases the probability of college admission and enrollment (Wei et al., 2019). Compared with students from high SES families, students with low family SES tended to underestimate their academic performance and were less likely to make optimal college choices (Wei et al., 2019). Children from lower SES families also had a higher possibility of experiencing depression compared to those from middle SES families (He et al., 2012). SES could influence children’s mental health (Jiang et al., 2018). It is necessary to address if SES has an influence on Chinese children’s hope.

Therefore, the present research had two principal aims: (1) to examine the measurement invariance of the CHS across SES in Chinese youth and (2) to compare the possible differences in hope between lower and higher SES youth. A baseline model was built to compare between the single-factor model and the hypothesized two-factor model before evaluating for measurement invariance. For the second aim, a test for raw scores and latent means of CHS was conducted to demonstrate the differences in hope.

## Materials and Methods

### Participants

Participants comprised 2081 students from six public high schools and six primary schools in three cities (Changsha, Chenzhou, and Loudi), Hunan Province, China. Each city has two high school and two primary schools. The age of the participants ranged from 8 to 17 years (*M* = 12.96, SD = 2.698). All questionnaires took approximately 10 min to complete and were collected simultaneously across the classes. Consent was obtained in written from both the school and the respondents’ guardians before beginning the survey.

One thousand nine hundred and thirty four valid questionnaires were returned with an effective response rate of 92.9%. The sample consisted of 975 females (50.4%) and 959 males (49.6%). The age of the participants ranged from 8 to 17 years (*M* = 12.96, SD = 2.686): 8-year-olds (*n* = 106), 9-year-olds (*n* = 182), 10-year-olds (*n* = 175), 11-year-olds (*n* = 100), 12-year-olds (*n* = 261), 13-year-olds (*n* = 245), 14-year-olds (*n* = 119), 15-year-olds (*n* = 324), 16-year-olds (*n* = 288), and 17-year-olds (*n* = 134). The Han nationality accounted for 91.73% for the sample (*n* = 1774), and other minorities accounted for 8.27%.

### Measures

#### Children’s Hope Scale

The CHS is a six-item self-report measure developed by Snyder et al. (1997) to assess hopeful thinking in children and adolescents. Responses range from 1 (none of the time) to 6 (all of the time). Items 1, 3, and 5 focused on assessing Agency thinking while items 2, 4, and 6 focused on assessing Pathway thinking (Snyder et al., 1997). We used a Chinese version of the scale in this study (Zhao and Sun, 2011).

#### Self-Esteem Scale

The Self-Esteem Scale was developed by Rosenberg (1965), which consists of 10 self-report items measuring global self-esteem. Each item requires participants to choose one of four response options [ranging from one (“strongly disagree”) to four (“strongly agree”)]. The scale has good reliability (Cronbach’s alpha coefficient ranging from 0.72 to 0.88; Gray-Little et al., 1997). Alpha coefficient was 0.791 in the present study.

### Family Socioeconomic Status

The family SES was measured by using the family monthly income and the parents’ education level. The family monthly income was reported by parents ranging from 1 (“<1000 *yuan* per month”) to 8 (“>10,000 *yuan* per month”). Parents’ highest attained educational level was categorized into six levels from 1 (illiteracy) to 6 (postgraduate education). Based on calculations for previous studies, the parents’ education level and family monthly per month were normalized and then added to equal the family SES scores (Plenty, 2018). Low SES and high SES groups were defined as the lower and upper 27% rule based on the family SES scores (*n* = 522 for each group) (Plenty, 2018).

### Data Analysis

A confirmatory factor analysis (CFA) and a multi-group CFA were conducted using Mplus 6.12 (Muthén and Muthén, 1998/2011). The maximum-likelihood parameter estimates with standard errors and a mean-adjusted Chi-square test statistic (MLM) estimator was chosen to estimate the parameters. Data analyses were carried out in three steps. First, the baseline model was built by a CFA method to examine the factor structure of CHS in the whole sample as well as separately by SES sub-group. The single-factor model and the hypothesized two-factor model established by Snyder (1994) were evaluated. Several fit indices were used to evaluate the model: the root-mean-square error of approximation (RMSEA), the standardized root mean squared residual (SRMR), the comparative fit index (CFI), and the Tucker–Lewis index (TLI). The model is considered acceptable if: RMSEA ≤ 0.08, SRMR ≤ 0.08, TLI ≥ 0.90, and CFI ≥ 0.09 (Hu and Bentler, 1999).

Factorial invariance of CHS across SES was tested by multi-group CFA methods in the second step. This consisted of a hierarchical set of four steps (Samuel et al., 2015): (1) configural invariance was tested with no constraints; (2) metric invariance was tested with constraints of equivalent factor loadings across SES; (3) the factor loadings were constrained and intercepts were set to be equal across SES (to test scalar invariance); and (4) strict invariance was tested by constraining factor loadings, intercepts of variables, and error variances to be equal across SES.

In the last step, the latent mean invariance was tested for. The factor scores were then calculated by setting the low SES group as the reference group and estimating the latent mean freely in the high SES group. Following this, the differences in factor scores between the two groups were examined utilizing a *t*-test.

The changes in CFI, along with the differences of Satorra–Bentler rescaled χ^2^ (△SBχ^2^), were chosen to evaluate the fit of nested models: a △CFI ≤ 0.010 supplemented by significant differences of △SBχ^2^ was considered evidence of invariance.

## Results

### Descriptive Statistics

The age, gender, and CHS scores of the subjects across SES groups are depicted in Table 1. The results show that these characteristics differ significantly between the two groups in the current sample with the higher SES group reported higher agency scores, pathways scores, and total scores than the lower SES group (Agency: *p* < 0.05, Cohen’s *d* = 0.14; pathways: *p* < 0.01, Cohen’s *d* = 0.20; total: *p* < 0.01, Cohen’s *d* = 0.19).

**TABLE 1:** Characteristics of study participants across socioeconomic status groups.

	**Lower SES (*n* = 522)**	**Higher SES (*n* = 522)**	***t*/χ^2^**	***p*-value**
**Characteristics**				
**Age (years)**				
Mean (*SD*)	13.92 (2.68)	11.97 (2.47)	12.22	<0.001
**Gender**				
Boys(%)	273(52.3%)	241(46.2%)	3.924	0.048
Girls(%)	249(47.7%)	281(53.8%)		
**CHS**				
Total	21.02 (5.06)	22.02 (5.54)	3.068	0.002
Agency	10.57 (2.75)	10.97 (3.02)	2.261	0.024
Pathways	10.45 (2.93)	11.05 (3.05)	3.26	0.001

*SES, socioeconomic status; CHS, Children’s Hope Scale.*

### Building the Baseline Model

Two competing models were evaluated to build the baseline model. In the one-factor model, all six items were loaded on a single factor. In the two-factor model, three items (items 1, 3, and 5) loaded on the Agency factor and other three items (items 2, 4, and 6) loaded on the Pathways factor. Models were tested separately using CFA for goodness of fit to the full sample, and the lower and higher SES groups.

The fit indices of CFA are showed in Table 2. For the two-factor model, all CFI and TLI values were >0.90; all the SRMR values were <0.08; the RMSEA values were <0.08 (full sample: 0.074, lower SES group: 0.968) with exception of the higher SES group (RMSEA = 0.085). However, all the fit indices were acceptable for the single-factor model. The difference between the two models was not significant (for full sample: △SBχ^2^ = 2.728, *p* = 0.099; for higher SES sample: △SBχ^2^ = 0.263, *p* = 0.608) with exception of the lower sample (△SBχ^2^ = 5.903, *p* < 0.05). Moreover, the standardized correlation coefficients between the two latent variables appeared quite high (full sample: *r* = 0.964; lower SES sample: *r* = 0.888; higher SES sample: *r* = 0.970). These results indicate that the single-factor model fits the data well for the full sample as well as for each SES group. The correlation coefficients between the two subscales and Self-Esteem Scale were calculated for the entire sample and each SES group (Table 3). The correlation coefficients between the Agency and the Self-Esteem scores did not show a significant difference compared to the correlation coefficients between the Path and the Self-Esteem scores (full sample: *p* = 0.412; lower SES sample: *p* = 0.910; higher SES sample: *p* = 0.101). Based on the following results, the one-factor model was chosen to serve as the baseline model.

**TABLE 2:** Goodness of fit indices for the baseline model.

	**SBχ^2^**	***df***	**RMSEA**	**SRMR**	**CFI**	**TLI**
**One-factor model**						
Full sample	95.344	9	0.070	0.040	0.957	0.929
Lower SES	22.410	9	0.053	0.031	0.973	0.956
Higher SES	38.626	9	0.079	0.041	0.953	0.921
**Two-factor model**						
Full sample	92.616	8	0.074	0.040	0.958	0.922
Lower SES	16.507	8	0.045	0.029	0.983	0.968
Higher SES	38.363	8	0.085	0.041	0.952	0.909

*SBχ^2^, Satorra–Bentler robust χ^2^; RMSEA, robust root mean square error of approximation; SRMR, standardized root mean-squared residual; CFI, comparative fit index; TLI, Tucker–Lewis index.*

**TABLE 3:** Person’s correlations between the CHS and Self-Esteem Scale scores.

	**Self-Esteem**		
	
	**Full sample (*n* = 1934)**	**Lower SES (*n* = 522)**	**Higher SES (*n* = 522)**
CHS	0.507^∗∗∗^	0.468^∗∗∗^	0.585^∗∗∗^
Agency	0.464^∗∗∗^	0.419^∗∗∗^	0.558^∗∗∗^
Path	0.450^∗∗∗^	0.415^∗∗∗^	0.510^∗∗∗^

*^∗∗∗^*p* < 0.001.*

Cronbach’s alpha coefficients and McDonald’s omega coefficients (0.736 and 0.745, respectively) were used for the full scale the sample. These two coefficients were 0.733 and 0.745, respectively, in the lower SES group and 0.767 and 0.775, respectively, in the higher SES group.

### Testing for Measurement Invariance of the CHS

The results of the measurement invariance tests across SES are shown in Table 4. The first step was to evaluate the configural invariance model (Model 1). The RMSEA, SRMR, TLI, and CFI indices indicate that the configural model yielded a good fit to the data. Next, the metric invariance model (Model 2) was assessed. Results showed that the corrected △SBχ^2^ = 20.22, *p* < 0.01, and the △CFI = 0.014, indicating that all factor loadings equally applied across groups did not hold. As such, we released item 5 which had the largest difference in factor loading between the two groups based on the results of Model 1. The partial loading invariance with one non-invariant loading (Model 3) yielded a good fit and scalar invariance was tested for (Model 4). Full scalar invariance was not observed: △SBχ^2^ = 12.804, *p* < 0.05, although the △CFI = 0.007. When we allowed the item 4 intercept to vary between the two groups, partial invariance was obtained: △SBχ^2^ = 9.319, *p* = 0.097, and △CFI = 0.002 (Model 5). Last, strict invariance was examined. Full strict invariance was observed: △SBχ^2^ = 10.174, *p* = 0.118, and △CFI = 0.003 (Model 6).

**TABLE 4:** Fit indices for CHS invariance across SES deprived from confirmatory factor analyses.

**Model**	**SBχ2**	***df***	**RMSEA**	**SRMR**	**CFI**	**TLI**	**△CFI**	**△SBχ2**	***p***
1. Configural invariance	60.266	18	0.067	0.036	0.963	0.938			
2. Metric invariance	80.486	23	0.069	0.053	0.949	0.934	0.014	20.220(2*vs*.1)	<0.01
3. Partial metric	66.006	22	0.062	0.040	0.961	0.947	0.002	5.740(3*vs*.1)	0.219
4. Scalar invariance	78.810	27	0.061	0.044	0.954	0.949	0.007	12.804(4*vs*.3)	<0.05
5. Partial scalar	75.325	26	0.060	0.043	0.956	0.950	0.002	9.319(5*vs*.3)	0.097
6. Strict	85.499	32	0.057	0.048	0.953	0.956	0.003	10.174(6*vs*.5)	0.118
7. Latent means	79.729	27	0.062	0.051	0.951	0.947	0.002	4.404(7*vs*.5)	<0.05

*SBχ^2^, Satorra–Bentler robust χ^2^; RMSEA, robust root mean square error of approximation; SRMR, standardized root mean-squared residual; CFI, comparative fit index; TLI, Tucker–Lewis index. Model 1 refers to the configural invariant factor model; Model 2 refers to the metric invariant factor model; Model 3 refers to a partial metric invariant factor model; Model 4 refers to a scalar invariant factor model; Model 5 refers to a partial scalar invariant factor model; Model 6 refers to the strict invariant factor model; Model 7 refers to a latent means invariant test.*

### Differences in Latent Means

We tested latent means invariance based on the partial scalar invariance model (Guo et al., 2009). It was not supported by △SBχ^2^ = 4.404, *p* < 0.05, and that △CFI = 0.002 (Model 7), indicating that latent means differ between the two groups. Next, according to independent-samples *t*-test, higher SES group had higher latent factor scores than lower SES group (*t* = 3.064, *p* < 0.01).

### Convergent Validation

Table 3 shows the correlations between the CHS and Self-Esteem Scale scores. Significant positive correlations were found across the three samples: full sample: *r* = 0.507, *p* < 0.001; lower SES sample: *r* = 0.468, *p* < 0.001; higher SES sample: *r* = 0.585, *p* < 0.001. The CHS displayed a stronger correlation with Self-Esteem in the higher SES group than lower SES group (*Z* = 2.618, *p* < 0.01).

## Discussion

The current study contributes to empirical research on the CHS in several ways. First, this study supports a better fit for a single factor model instead of the two-factor model proposed by Snyder (1994). This finding is consistent with some previous studies (Bickman et al., 2010; Savahl et al., 2016). However, other previous studies have found a reasonable fit of the hypothesized correlated two-factor model to the data (Atik and Kemer, 2009; Marques et al., 2009; Jovanović, 2013; Pulido-Martos et al., 2014). This may suggest that the CHS performs as a different construct in different cultural contexts.

Second, according to our knowledge, we provided the first evidence for the fit of the CHS single-factor model across SES using a multi-group CFA method. The presence of configural invariance suggested that in both lower and higher SES Chinese youths, the CHS may be assessing the same latent construct – a common frame of reference for hope-related thinking be shared across the two groups. The findings of partial metric and scalar invariance in this study indicated that the mean differences in the latent factor cannot fully explain the observed mean differences on the CHS items. It is therefore necessary to be cautious when comparing differences in hope between groups with different SES, when measured using the CHS.

We found that one factor loading (item 5) and one intercept (item 4) were not invariant across the two SES groups. Item 5 (“I think the things I have done in the past will help me in the future”) exhibited loading non-invariance between lower and higher SES groups. Factor loadings are structural regression coefficients, which represent the magnitude of expected change in the observed variables for every unit of change in the latent variable. They reflect the degree to which differences among participants’ responses to the item arise from differences among their levels of the underlying construct that is being assessed by that item. Thus, our results suggested that there is a non-identical relationship between Agency and the participants’ responses to item 5 across SES. Item intercepts are the origin or starting value of the scale that the factor is based on. Non-invariance of intercepts for Item 4 (“When I have a problem, I can come up with lots of ways to solve it”) may be indicative of potential measurement bias. This suggests that family SES influences the way that participants are responding to items and that participants with lower SES are systematically rating item 4 higher than those with higher SES. Our results regarding the latent mean differences across SES show that the latent means were lower in Chinese youth with lower SES than in those with higher SES. This was consistent with the results from the comparison of raw scores conducted by *t*-test analysis. Given the non-invariance of latent means, and the significant differences in latent means and raw scores on the CHS scale, we must be careful when we use this scale to measure hope-related thinking in children and adolescent growing up in significantly different family SES conditions. This finding supports the family investment theory. According to this theory, the family’s SES reflects a difference in access to real or potential resources (Conger and Donnellan, 2007). Parents from high SES families can offer more social and economic resources for the future development of their children (Conger and Donnellan, 2007), which makes for a more valid and clearer future goal orientation and a higher level of hope (Griskevicius et al., 2011; Schroder et al., 2011). Whereas those from low SES families can invest limited resources for children’s development, which causes them to have fewer opportunities to achieve their goals and affects their perception of future goals (Conger and Donnellan, 2007; Griskevicius et al., 2011; Schroder et al., 2011). Our results also provided indirect support for our previous study (Yin et al., 2019), in which family SES was found to have a significant effect on the developmental trajectory of hope among late-adolescents.

We found evidence for substantial convergent validation in the whole sample and two SES groups, which is consistent with previous studies (Snyder et al., 1991a, 1997; Jovanović, 2013). This suggests that children with higher levels of hope might report feeling more positively about themselves. Laboratory evidence has also indicated that one’s self-esteem can increase by a manipulation of increasing one’s sense of successful goal pursuits (Snyder et al., 1996). Interestingly, the CHS had a stronger convergent validation for participants with higher SES than ones with lower SES. This indicates that participants’ SES could be considered a moderator when investigating the correlations between the CHS and other positive constructs such as Self-Esteem, optimism, and self-efficacy.

It is important to acknowledge that the present study contains several limitations. First, there are multiple ways of measuring SES (Cirino et al., 2002). Our results, which provide the first examination of measurement invariance of CHS across SES, require replication in future research that may include alternate measures of SES. Second, the two SES groups in our study differed significantly in terms of gender and age. Previous studies, however, have reported CHS scores that do not differ by age (Snyder et al., 1997; Valle et al., 2004) or gender (Marques et al., 2009; Jovanović, 2013). Past studies examined measurement invariance across gender on the CHS among Mexican American adolescents (Edwards et al., 2007) and Serbian adolescents (Jovanović, 2013), providing evidence to support measurement invariance across gender. Thus the robustness of our results might not be affected by gender and age despite gender and age differences across SES groups. Another limitation of the current study is the cross-sectional study design that measures at one exact point in time and does not allow for the ability to capture changes in hopeful thinking across time. A survey to determine the longitudinal measurement invariance of CHS is needed. The last limitation concerns the generalization of our results as the sample population was collected from four cities in the Hunan Province. It is unknown whether our findings will demonstrate reliability and validity in a different sample from another area in China.

In sum, this is the first study to demonstrate measurement invariance across SES on the CHS, which aims to be a meaningful contribution to the measurement of the children’s hope. It is necessary to be cautious when comparing differences in hope measured by the CHS between groups of participants from different SES families because of partial metric and scalar invariance. Future research may consider utilizing longitudinal measurement invariance of the CHS. Furthermore, our findings provided more robust statistical evidence that children and adolescents from higher SES backgrounds showed higher levels of hopeful thinking compared with those from lower SES backgrounds. More research is called for to further examine the relationship between family SES and children’s hope.

## Data Availability Statement

The datasets generated for this study are available on request to the corresponding author.

## Ethics Statement

The studies involving human participants were reviewed and approved by the Hunan Agriculture University. Written informed consent to participate in this study was provided by the participants’ legal guardian/next of kin.

## Author Contributions

ZL was mainly responsible for the overall conception and design of this study. HL wrote the manuscript and carried out the statistical analysis. ZW, ZP, and YY carried out the investigation and data collation work.

## Conflict of Interest

The authors declare that the research was conducted in the absence of any commercial or financial relationships that could be construed as a potential conflict of interest.
